# The retroviral RNA dimer linkage: different structures may reflect different roles

**DOI:** 10.1186/1742-4690-1-22

**Published:** 2004-08-18

**Authors:** Jane Greatorex

**Affiliations:** 1 Division of Infectious Diseases, Dept. of Medicine, University of Cambridge, Addenbrooke's Hospital, Hills Road, Cambridge, CB2 2QQ, UK

## Abstract

Retroviruses are unique among virus families in having dimeric genomes. The RNA sequences and structures that link the two RNA molecules vary, and these differences provide clues as to the role of this feature in the viral lifecycles. This review draws upon examples from different retroviral families. Differences and similarities in both secondary and tertiary structure are discussed. The implication of varying roles for the dimer linkage in related viruses is considered.

## Introduction

With relatively few genes compared to many other virus families, the retroviridae have evolved over the millenia to maximise the functions of their RNA genome. The genome serves as a versatile template from which various proteins can be translated by the use of splicing and by translational flexibility using scanning, IRES and frameshifting. It is also an RNA molecule capable of interacting with itself, and cellular and viral proteins. By these means, from an RNA around 7 – 12 kilobases long, the retroviridae have evolved to infect a wide range of species and cell types.

A unique characteristic of retroviral genomes is the fact that they are dimeric. The reasons for this are as yet unclear, and are discussed below. In brief, it is thought that the diploid genome allows template switching during reverse transcription and may be linked to recombination in some viruses. It may also play a role in translation of proteins and packaging of the RNA.

Much of the work on the nature, structure(s), and role of the dimer linkage has been based on Human Immunodeficiency Virus Type 1, and this has been recently reviewed ([1] and Russell *et al *this issue [2]). Whether or not HIV-1 is a representative model for other retroviruses is open to debate. However, there have been important contributions from investigators studying other retroviruses. They have shown both similarities with the HIV-1 motifs, and also, importantly, differences. The fact that distinct RNA structures are used by different retroviruses to perform the same purpose, namely to link their two RNA molecules, tells us something very important. For these viruses, whatever organism or cell they are infecting it has been advantageous to evolve to do so with a double complement of genome in their virion particles. However, diploidy may be used to benefit the virus in a number of ways and for different viruses the priorities may vary. This review will attempt to draw on several examples from viruses other than HIV-1, whilst of necessity drawing comparisons with the latter.

## The dimeric genome

Retroviruses were discovered at the beginning of the 20th century [3,4]. The unique nature of their genome was first discovered in the 1960s [5,6] but the actual dimeric genomes were elucidated, and visualised by electron microscopy, a decade later [7,8]. Bender and colleagues extracted the RNA from several different retroviruses and examined it by electron microscopy under denaturing conditions. The RNA appeared to be joined at a discrete point, termed the dimer linkage site (DLS). Using bromodU to label the RNA at one end, they were able to show that the molecules were joined at their 5' ends [9,10]. Under less stringent conditions the genomes can be demonstrated to interact along their lengths [11] and it is this that probably contributes to confusing reports on the exact location of the primary DLS in different viruses.

RNA dimerisation in the primate lentiviruses, predominantly HIV-1, has subsequently been extensively studied [1], yet little has been published on this process in the non-primate lentiviruses. Early studies of rapid harvest virions of the prototype lentivirus, Maedi Visna virus (MVV), identified viral RNA with a Svedberg coefficient of 35S immediately post-budding, which increased with time to 70S. It is possible that weakly interacting dimers formed during RNA encapsidation may have been denatured during purification, however these observations are supportive of a progression from monomeric to dimeric RNA associated with viral maturation [12].

Since 1990 [13] it has been possible to study *in vitro *the RNA elements involved in the dimer linkage first observed by EM. It was shown that RNA transcripts comprising sequences from the 5' end of the viral genome would migrate as two species of RNA when subjected to electrophoresis. By this means many subsequent studies were able to focus on isolating the elements and structures involved in dimerisation, and to investigate the role of the viral structural proteins in this process.

## Multiple functions for the dimeric genome?

As yet investigators have not been able to agree on a distinct role for the dimer linkage. The fact that it is conserved amongst the retroviridae does not guarantee that its role will be the same in all retroviral families. The following section of the review will endeavour to explore some of the proposed roles, and examine the evidence from different retroviruses.

## The dimeric linkage and recombination

Several studies have demonstrated that, in HIV-1 and MLV, the dimer linkage serves as a "hotspot" for recombination [14,15]. It is an obvious hypothesis, that in viruses which are known for their hypervariability, there exists the capacity to jump from one RNA molecule to another. Researchers have compared dimerising to non-dimerising controls, and the frequency and distribution of template switching. Templates containing the dimerisation site had a 4-fold higher transfer efficiency than the non-dimerising control [14]. This result implies that recombination would occur preferentially at the site where the RNA molecules were in close proximity. In the case of HIV-1, whilst it has been shown that template switching is facilitated by template homology [16], it has also been demonstrated that recombination can occur between viruses of different subtypes which might have different dimer initiation sequences (DIS) [17]. Bearing in mind the fact that the genome is linked at other sites besides the DIS [11], it seems probable that other hot spots for recombination exist.

Interestingly, it has been suggested that the nucleocapsid protein (NC) promotes or stimulates the strand transfer reaction. As will be discussed below, NC and the precursor Gag protein both bind the RNA close to the DIS in HIV-1. In addition, there is evidence that the presence of a dimer in the virus particle facilitates the first strand-transfer reaction of reverse transcription [18].

Work in our laboratory has shown that the Maedi Visna Virus DIS is centred on a helix terminating in a GACG tetraloop between positions 281 and 300 in the viral genome; a region which is highly conserved between the ovine and caprine lentiviruses (Monie, personal communication, see Figure 3d). Intriguingly, this structure shows homology with structural motifs in the *Alpha- *and *Gammaretroviruses*, but not with DIS regions identified in the primate lentiviruses. Within the *Alpha*- and *Gammaretroviruses *GACG tetraloops are involved in the packaging of viral RNA [19,20] and whilst not a component of the core M-MLV DIS motif [21], they may contribute to the process of dimerisation and the stability of the resultant dimer [22]. Importantly, it is possible to form heterodimers between transcripts from these viruses containing the GACG tetraloops and between MVV and M-MLV (personal observations). This raises parallels with recent studies of the dimerisation of murine leukaemia viruses and Harvey Sarcoma virus in which GACG tetraloops were found to regulate inter-species RNA heterodimerisation [23], whilst other linkage elements were postulated to mediate homodimerisation.

Recombination, and the genomic variability it confers cannot be the sole function of the dimeric genome, since retroviruses with highly conserved genomes and little sequence variability such as HTLV-1 [24] are also dimeric.

## Translation and packaging?

**Figure 1:**
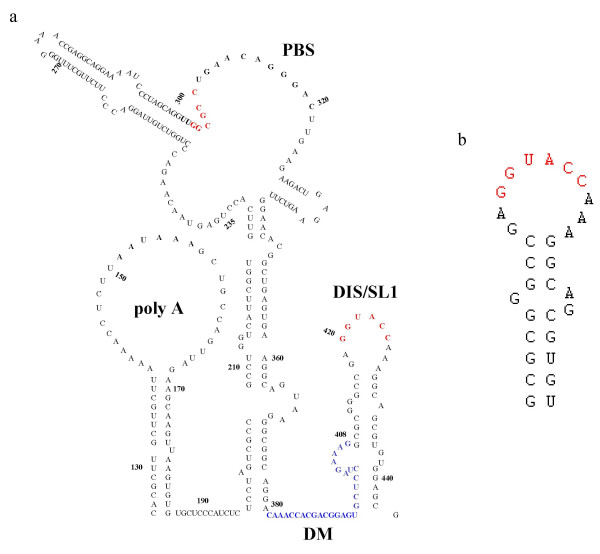
Structure of the HIV-2 leader region. 1a. Secondary structure model of the HIV-2 leader region based on mfold predictions. Indicated are the putative dimer linkage sites (in red). Also highlighted is the DM region defined as being critical for packaging [28], in blue). 1b. The effect of the DM deletion on the SL1/DIS stem loop. The stem is truncated and the internal bulge altered in approximately half the predicted structures.

Another possible role is that of the dimer linkage acting as a switch, its presence permitting or restricting the packaging of RNA. In HIV-2 two regions were originally suggested as dimer initiation sites, one analogous to the palindromic sequence identified as the principal DIS in HIV-1 (termed SL1), one close to the PBS [25-27]. Recently, a region upstream of SL1 (also called the DIS, see Figure 1a) was identified as being critical for packaging [28]. An extensive deletion analysis of the 5' leader of HIV-2 was carried out, and removal of nucleotides 380–404 (HIV_ROD_), termed the DM region, rendered the virus severely packaging deficient. The mutation had been designed based on the mfold [29,30] prediction, that removal of these sequences would disrupt the SL1 structure and hence dimerisation (Figure 1b). *In vitro *studies using RNA transcripts comprising the leader region with and without the DM deletion, reveal that it does, indeed, render the viral RNA monomeric (personal observations). Using antisense oligonucleotides, another group have demonstrated that this region may, in fact, play a role in the dimerisation process itself [31]. By free energy minimisation this region is predicted to be unstructured, so it is not clear how the RNAs would interact with one another. In addition, whilst the SL1/DIS sequence is conserved amongst HIV-2 and SIV sequences in the database, that within the DM region is less so, and the substitutions which exist would affect the auto-complementarity of the sequence.

**Figure 2:**
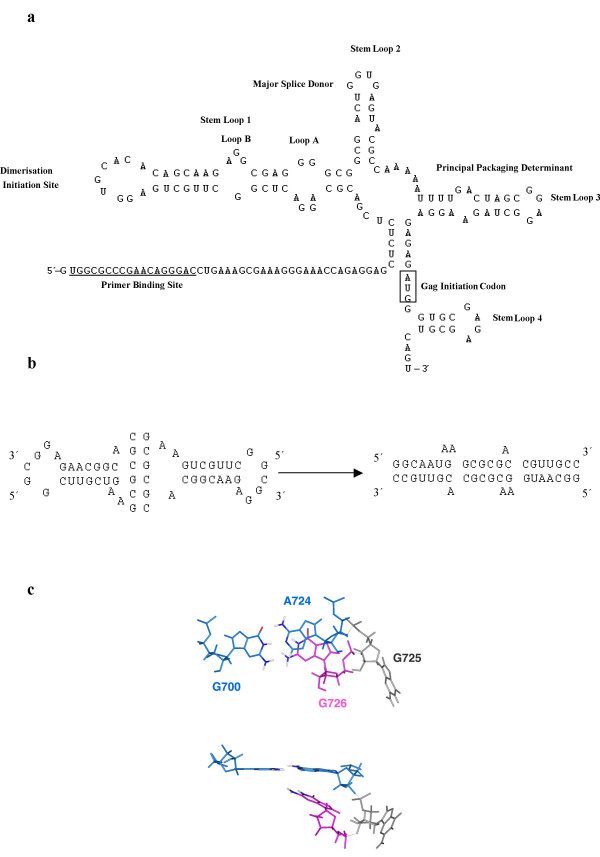
Structure of the key elements involved in HIV-1 RNA dimerisation. 2a. Secondary structure model of the packaging signal of HIV-1_Lai _([64] [65]), containing the principal DLS. 2b Proposed sequence of the RNA dimerisation process in HIV-1_Lai_. The initial kissing hairpin interaction (including loop B) followed by formation of the extended duplex ([1]). 2c. Loop B, one of the critical elements in the dimer interaction. The flexibility of this internal loop allows the duplex to form ([44]).

**Figure 3:**
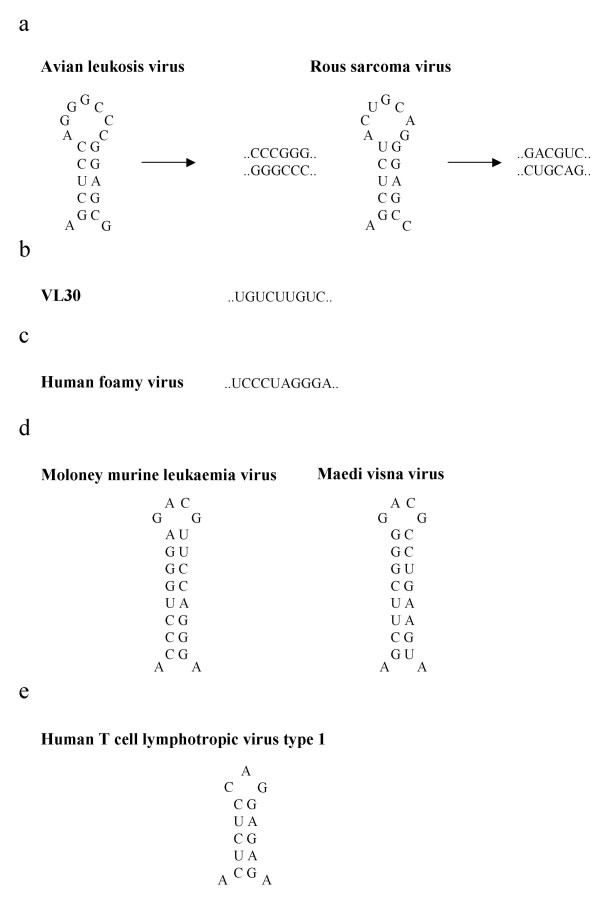
Dimer linkages of the retroviridae (excluding the lentiviruses). 3a. Loose and tight dimers ([51]). 3b. Imperfect repeats ([66]). 3c. Palindromes ([38]). 3d. GACG loops ([23]). 3e. CAG tri-loops (see Figure 4).

**Figure 4:**
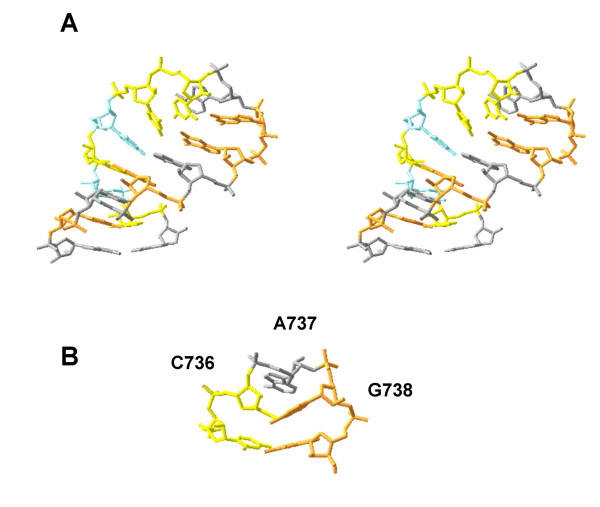
Proposed tertiary structure of the HTLV-1 dimer linkage. 4a Stereoview of 3D molecular modelling of a potential structure of the HTLV-1 DIS from nucleotide A730 to A744 using JUMNA ([61]). 4b. Close up of the terminal loop. Bases are coloured as follows: adenine, grey; cytosine, yellow; guanine, orange; and uracil, cyan.

One of the key differences between HIV-1 and HIV-2 replication is their modes of packaging [32]. Whilst the Gag protein of the former captures the genomic RNA *in trans*, the latter uses predominantly a *cis *mechanism. One might postulate therefore, that, if retroviruses must package a dimeric genome, it is critical in the case of HIV-2 that the genome is dimeric before interacting with the Gag polyprotein. Hence, the effect of mutations in the DM region may be to render the RNA monomeric and thus to severely impair packaging.

It is attractive to speculate that the reason packaging itself is not affected by DIS mutations to the same degree in HIV-1 [33] is this difference in protein:RNA interaction. If the RNAs can interact at points other than the principal DIS over time, then perhaps the *trans *mechanism is less dependent on a high affinity dimer linkage?

## Particle maturation and viral infectivity

A recurring observation amongst investigators is the fact that mutation or deletion of dimer linkage sites causes viral infectivity to be decreased [33]. One explanation for this might be that a dimeric genome is a prerequisite for maturation of virus particles. Certainly, immature HIV-1 particles are non-infectious, and viruses with their DLS mutated have been demonstrated to form only immature particles [34].

The DLS of Human T Cell Lymphotropic Virus Type 1 (HTLV-1) was identified as a 14-nucleotide sequence just downstream of the splice donor [35]. Removal of this region from the leader sequence rendered the RNA monomeric *in vitro *[24,36]. When this deletion was introduced into the wildtype genome sequence, the only viral replication defect that was observed, following transfection and subsequent infection, was that of impaired infectivity [37]. Likewise, a similar effect was observed when the DLS of Human Foamy Virus was mutated [38].

Parent *et al *showed that if the RNA of Rous Sarcoma Virus (RSV) was engineered so that it was monomeric, the virus was non-infectious [39]. Interestingly, this group suggested that it might be a difference in localisation of structural proteins and RNA affecting subsequent dimer formation and viral infectivity [40]. This is an area that has not been explored to any extent. Also working with RSV, Bieth and colleagues found that, in an *in vitro *system, dimer formation appeared to inhibit synthesis of the Gag polyprotein precursor [41].

## Structure of the dimer linkage

Undoubtedly the best defined dimerisation structure is that involved in the dimer linkage of HIV-1. The discovery of the sequences involved, the subsequent description of the RNA:RNA interaction, and the elucidation of the tertiary interaction are described elsewhere [1]. The initial interaction between the two RNAs appears to be a kissing loop interaction (similar to that seen in the regulation of plasmid replication, [42]) followed by annealing of the two RNAs into an extended duplex (Figure 2b). The sequences contained within the palindrome are remarkably conserved. Using an *in vitro *selection system it has been possible to demonstrate that the DIS has evolved to satisfy both constraints for optimal dimerisation affinity, and the potential to homodimerise [43]. The dimer linkage is found at the terminal end of Stem Loop 1 (SL1) within the packaging signal region of HIV-1 (Figure 2a). The tertiary structure of the whole SL1 RNA has been determined [44,45] and the structures have helped to determine exactly how the RNAs interact with one another. A number of elements appear to be critical for the dimer interaction: flanking purines and central nucleotides in the palindromic sequence [46] and loop B [47-49]. The tertiary structure of the latter has been described (Figure 2c), and there is some debate as to how flexible this internal loop might be. However, work by Borer *et al*, examining the interaction of NC with elements of the packaging signal, of which loop B is one, showed that, in fact, both structures might exist, the flexible one allowing NC binding at high affinity [50]. There are similar linkages in other retroviruses. The Avian Leukosis Viruses also interact firstly in a kissing hairpin manner, and then form an extended duplex (Figure 3a, [51]).

Palindromes remain a theme throughout many of the viruses investigated to-date. As already mentioned, the DIS of HIV-2 is less well defined than that of HIV-1. Whilst there is a palindromic sequence at the top of a stem loop structure that closely resembles the HIV-1 DIS (see Figure 1), there are other regions which have also been demonstrated to be important for dimer formation [25,26]. Other viruses with palindromic sequences as their DLS include HFV (Figure 3c) and MoMLV. In the case of MLV there are other sequences and structures which may play a role in dimer formation, including the GACG tetraloops mentioned previously [52]. The tertiary structure of this stem loop is the only proposed dimer linkage element yet to be determined in a retrovirus other than HIV-1 ([53]). RSV and VL30, also have imperfect repeat sequences in their dimer linkages [54,55] (Figure 3b).

Recent work by Monie and colleagues [36] describes the potential tertiary structure of the HTLV-1 dimer linkage, capped by a novel CAG tri-loop (Figure 3e and Figure 4). This tri-loop is formed by an unusual C:synG base pair closing the loop. Other similar loops have been described, in the domain IIId terminal loop of the hepatitis C virus internal ribosomal entry site (IRES) [56] and in stem loops required for initiation of transcription within the Bromoviridae [57]. Although sequence heterogeneity between HTLV-1 isolates is rare, distinct mutations identifying individual strains can be identified. Of 101 HTLV-1 sequences identified from the EMBL database, 90 showed sequence homology with HTLV-1_CH_, the strain used in the study. The other 11 sequences comprised three different variants. Eight contained a deletion of C736 (see Figure 4), two possessed the substitution A737G, and one possessed the substitution C733U. The substitution mutants have minimal impact on regional secondary structure, while the deletion may induce formation of a CAGG tetraloop. Interestingly, the A737G mutation possesses homology with 150 deposited HTLV-II sequences, suggesting a conservation of the DIS between HTLV-I and -II.

## Conclusions

The retroviral RNA genome structure does not stay static during the course of transcription, translation and ultimately packaging. Various investigators have suggested that this constantly changing RNA structure plays an intimate role in the viral replication [58-61]. It seems possible that linkage of the two RNA molecules constituting the genome is integral to the changes in RNA structure. As described in the article above, the dimer also acts as a mechanism for promoting recombination; may be a signal for packaging to occur; may be an inhibitory signal; may direct processes to occur in specific cellular compartments; and lastly, may be capable of interacting with cellular proteins.

*In vivo *data has revealed just how important an intact dimmer linkage may be to a retrovirus. For instance, there are intriguing differences in the effect of dimer mutations on viral infectivity depending on the cell type being infected [62]. What the significance of this might be in the context of a viral infection is, as yet, unclear. The importance of the dimer linkage is perhaps most clearly exemplified by the observation that a patient infected with a viral isolate having a defective DLS, had a low viral load. The subsequent switch in the predominant virus to that with a competent DLS coincided with a rise in viral load [63]. One can speculate that, at least in the case of HIV-1, only those viruses with a whole, optimised dimer linkage are capable of efficient infectivity. For the purposes of examining the role of retroviral RNA dimer sequences in the context of animal models, the non-human retroviruses, including the non-primate lentiviruses will be of great importance.

To sum up, retroviral dimeric genomes are linked by a variety of RNA structures, including kissing hairpins, GACG tetraloops and unusual CAG-tri loops. The differences in these interactions, and when or where they occur, may reflect different demands upon this unique feature, and highlight the elasticity of the RNA genome.

## Competing interests

None declared.
